# Predictors of timeliness of vaccination among children of age 12–23 months in Boricha district, Sidama region Ethiopia, in 2019

**DOI:** 10.1186/s12887-023-04234-4

**Published:** 2023-08-19

**Authors:** Berhan Tsegaye Negash, Yoseph Tediso, Amanuel Yoseph

**Affiliations:** 1 Department of Midwifery, College Medicine and Health Science, Hawassa University, Hawassa, Sidama Ethiopia; 2 Department of Public Health, College of Medicine and Health Science, Hawassa University, Hawassa, Sidama Ethiopia

**Keywords:** Timeliness, Vaccination, Children, Sidama

## Abstract

**Background:**

Traditional measurement of vaccine coverage can mask the magnitude of timely uptake of vaccine. Hence, the optimal measurement of timeliness is unclear due to variations in vaccine schedule among countries in the world. In Ethiopia, Oral Polio Virus (OPV), Pentavalent, Tetanus, H. influenza type B, Hepatitis B, and Pneumonia-Conjugate Vaccine (PCV) are basic vaccines which are taken at birth, six weeks, ten weeks, and fourteen weeks respectively. Despite its importance, information is scarce about on-time vaccination in the study area. Therefore, this study aimed to assess prevalence and factors associated with on-time vaccination among children of age 12–23 months in Boricha district, Sidama Ethiopia, in 2019.

**Methods:**

A community based survey was conducted in Boricha district, Sidama region Ethiopia from January 1–30 in 2019. Study participants were selected using stratified multistage sampling technique. Kebeles were stratified based on residence. First, Kebeles were selected using random sampling. Then, systematic random sampling was employed to reach each household. Data were collected using structured and interviewer administered questionnaire. Logistic regression analysis was employed to identify factors associated with timely vaccination. Then, independent variables with *p*-value < 0.25 in COR were fitted further into multivariate logistic regression analysis model to control the possible cofounders. AOR with 95% CI and *p*-value < 0.05 was computed and reported as the level of statistical significance.

**Results:**

From a total of 614 study participants, only 609 study participants have responded to questions completely making a response rate of 99.2%. Prevalence of timeliness of vaccination was 26.8% (95% CI: 25, 28) in this study. Factors like children of women with formal education (AOR = 5.3, 95%CI,2.7, 10.4), absence of antenatal care visit (AOR = 4.2,95%CI, 1.8,9.8), home delivery (AOR = 6.2,95%CI,4.0,9.3), lack of postnatal care (AOR = 3.7,95%CI,1.1,13.3), and lack of information about when vaccines completion date (AOR = 2.0, 95% CI,1.13,3.8) were factors influences timely vaccination among children of age 12–23 months.

**Conclusion:**

Prevalence of on-time vaccination among children of age 12–23 months is lower than national threshold. Therefore, sustained health education on vaccination schedule and reminder strategies should be designed and implemented. Furthermore, maternal and child health care services should be enhanced and coordinated to improve on-time uptake of vaccine.

**Supplementary Information:**

The online version contains supplementary material available at 10.1186/s12887-023-04234-4.

## Background

Globally, the performance of vaccination programs has been assessed through vaccine coverage indicators. It is the proportion of children who have received a vaccine by a benchmark age, or number of doses of vaccine for a particular antigen in the specific age group regardless of the timing of administration [1]. Although vaccination coverage and defaulting are useful metrics, there can be substantial disparities between these measures and measures that consider timing of vaccine administration [2]. Vaccination coverage and timeliness of vaccine are related but separate issues. High level of vaccine coverage can sometimes mask the low level of vaccine timeliness [3]. Maintaining high-quality expanded immunization program is challenging, with non-attendance and delays to vaccination appointments in developing countries [4, 5]. Consequently, timely vaccination is the pivotal action during infancy, as trans-placental immunity decreases rapidly after birth [6].

Based on the World Health Organization (WHO) report, vaccine coverage indicator has failed to show on-time vaccine coverage [7]. According to WHO recommendation, timely vaccination indicates when a child completes age-appropriate vaccination [8]. Several studies also revealed that despite high vaccine coverage in most countries, there is substantial gap on the timely administration of childhood vaccination with substantial delay in age-appropriate vaccine [9–11].

Globally, the Expanded Program of Immunization (EPI) agenda was planned by applying WHO standards to give the primary vaccination series to at least 90% of children [12]. Ethiopia launched the EPI program in 1980. Currently, vaccines are provided for 11 antigens at birth, six weeks, ten weeks, fourteen weeks and nine months [13]. Although vaccines are provided freely in Ethiopia, the total vaccine coverage was still 49.9% in 2016 [14]. This is well below the target of 95% coverage of fully immunized infants set by the Health Sector Transformation Plan (HSTP) by 2020 [15, 16]. Regarding the overall timeliness of vaccination, the coverage was increased from 18% in 2014 to 22% in 2016 [17]. Particularly, Diphtheria Tetanus Pertussis (DTP_1_) (63.8%), Oral Polio Vaccine (OPV_1_) (63.1%), and measles (68.5%) were delayed for more than one month beyond WHO recommended schedule [18].

Currently, the timing of vaccine administration has received increasing attention when the level of vaccination coverage is close to that needed for protective herd immunity [19, 20]. Vaccine timeliness is critical in Sub-Saharan African countries since most of the causes of child deaths are vaccine preventable diseases [5]. It is used to observe active response, minimize individual vulnerability, and prevent disease outbreaks within societies in a given country [21].

Previous studies have revealed that untimely vaccination of measles poses a threat to susceptible children [2, 3]. Besides, previous studies highlighted that vaccine timeliness is a crucial indicator to understand population-level disease susceptibility [15, 16]. Therefore, age appropriate vaccine coverage has to be achieved to control and eliminate vaccine-preventable infectious diseases [19, 22, 23].

Previous study in Ethiopia showed that socio-demographic, maternal health service and health institution factors were associated with delay or early vaccination of children. For example, women’s educational status, wealth index, and women who used maternal and child health services were positive predictors of on-time vaccination. On the contrary, older caregivers, distant health institutions,and vaccinations at health post were protective against on-time vaccine coverage [24]. Marked child immunization differentials are also observed by community and ethnic group effects suggest that further targeting of health activities could be efficient and effective [25]. Although studies have measured the full vaccination coverage, studies on timely vaccination are rare in Sub- Saharan Africa [26, 27]. Specifically, a few previous studies were conducted about timeliness of vaccination in different parts of Ethiopia; most of them were conducted either at urban settings or at the national level [24, 28–30]. Local contextual policy planning should be mandatory for achieving the national expanded programe of immunization target. However, information is limited about the timeliness of vaccinations in Sidama, Ethiopia. Therefore, this study aimed to assess the prevalence of incomplete immunization status and factors among children 12–23 months of age in Boricha, South Ethiopia, in 2019.

## Methods

### Study design, period and setting

Community based cross-sectional study was conducted in Boricha district, Sidama region, Ethiopia from January 1–30, 2019. Boricha is one of 36 districts in Sidama region, in Ethiopia. It is 32 km away from Hawassa, the capital city of Sidama regional state. It is divided into 43 Kebeles (lowest administrative units in Ethiopia). There are 39 rural and 4 urban Kebeles in Boricha. Based on 2007 Ethiopian Central Statistical Agency (CSA) report the total population of the district was estimated as 317,605 in 2011. From this population, 16,452 were children of age 12–23 months. There were 10,132 infants in the district. A total of 50 public health institutions have provided vaccine freely in Boricha. These include 1 district hospital, 10 health centers, and 39 health posts. The health service coverage of the district was estimated as 85% for the total population.

### Population

All children in the age range of 12–23 months who had at least single exposure of vaccination and permanently resided (lived for more than six months) in Boricha district were the source population. Children aged 12–23 months had at least single exposure a vaccination, lived in the selected households of selected Kebeles, and presented during data collection period were the study population in this study. All children of age 12–23 months, who were permanent resident of selected Kebeles, and whose mother / Guardians / were voluntary for consent, were included. On the contrary, children in 12–23 months whose mothers were seriously ill and, whose mothers or legal guardian refused to give consent were excluded from this study.

### Sample size determination and sampling technique

The minimum sample size was calculated using a single population proportion formula. Furthermore, sample size determination considered the following key factors: Power, precision, baseline proportion of timeliness of vaccine, quality and cost of the study. Therefore, sample size was determined using the following assumptions: Proportion of timely vaccination of 41.2% from Ethiopian Demographic Health Survey (EDHS) analysis of vaccine timeliness of South Ethiopia [31], 95% confidence level, and 5% margin of error, a design effect of 1.5 and 10% non-response rate. Thus, standard Cochran formula (n = z^2^pq/d^2^) was used to compute the initial sample size. Plugging the values of above assumptions into formula, n = 1.96^2^*(0.41) (0.59)/ (0.05)^2^. As a result, we have calculated as 372 sample size. Next, by considering sample size compensation factor of design effect of 1.5 and adding 10% non-response rate. The final sample size was computed as 614 for this study. The calculated sample sizes for factors associated with vaccine timeliness were lower compared with sample size calculated to prevalence of vaccine timeliness. Therefore, data were collected on a total of 609 children. Multi-stage stratified sampling strategy was employed to get actual study participants in the study. First, all Kebeles were stratified using residence as urban and rural Kebeles. Then, sample Kebeles were selected proportionally within each stratum. From a total of 39 Kebeles, 1 urban and 9 rural Kebeles were selected. List of households with children aged 12–23 months was utilized as the sampling frame from the registration book of health extension workers in each health posts. Then, systematic random sampling was applied to select sample households. Finally, simple random sampling technique was utilized to select a child for households which contain more than one child. If no one answered at a household, a revisit was conducted before the household was labelled as a non-respondent.

### Data collection tools and procedures

Data were collected using structured, and interviewer administered questionnaire. The questionnaire was adapted from EDHS questionnaire. It included the following three parts: socio-demographic characteristics, information about vaccination completeness, and information about maternal health service utilization. Data were collected by 10 BSC Nurses who are fluent Sidama-afoo; local language. Two health officers supervised the data collection process daily. Problems, which were raised during data collection period, were solved through discussion at the end of every day.

### Study variables

Timeliness of vaccination is the outcome variable in this study. It is a binary outcome variable. A woman/guardian/ was asked to show vaccine card or/and recall the time of birth of the child and her child vaccination appointment. Then, the vaccine schedule was compared with the vaccine card for inconsistency. If there was delay or early vaccination from WHO standard recommendation, timely vaccination was absent, and recoded as ‘0’, otherwise,’1’. The explanatory variables included in the analysis of this study were socio-demographic characteristics, vaccine information related factors, and maternal health services utilization. Socio-demographic variables were the following factors: Age, educational status, occupation, place of residence, marital status, monthly income level and family size. Child and maternal health service related factors consisted antenatal visit, place of delivery, postnatal care, child birth order and sex of a child. Information related variables were: Awareness vaccine preventable disease, vaccine protects from illness, session needed for full vaccination, age of children to begin vaccine, age of children to complete vaccine, importance of vaccine for child health and vaccine can cause health problem.

### Operational definitions

#### Full vaccination

Was defined as the child vaccination status once an infant has received all recommended vaccines included in the national schedule: A dose of Bacilli Chalmette Guerin (BCG); three doses of Oral Polio Vaccine (OPV); three doses each of Pentavalent and Pneumococcal Conjugate Vaccine (PCV); one dose of Inactivated Polio Vaccine (IPV); two doses of rotavirus and one dose of measles vaccines by the age of 12 months [32, 33].

#### Vaccine timeliness

Was defined as vaccine dose administered within four days prior [27, 33] and within four weeks after the recommended age-specific vaccine according to national immunization schedule [23, 27, 34]. Table 1 of this study indicates that on-time full vaccination was also defined as all vaccine doses administered within four days prior [27, 33], and within four weeks after the recommended age specified in the national immunization schedule. Otherwise, it was not considered as on-time full vaccination if at least one vaccine dose was given early, late or missed at all [27, 34, 35] (see Table 1).

**Table 1:** Routine immunization schedule, in Ethiopia, 2014

BCG	Tuberculosis	At Birth
Pentavalent	Diphtheria, Pertussis, Tetanus, H. influenza type b, Hepatitis B	6, 10, 14 weeks
OPV	Polio	At birth, 6, 10, 14 weeks
Pneumonia-conjugate Vaccine (PCV)	Pneumonia	6, 10, 14 weeks
Rotarix (rotavirus vaccine)	Rotavirus	6, 10 weeks
BCG	Tuberculosis	At Birth
Pentavalent	Diphtheria, Pertussis, Tetanus, H. influenza type b, Hepatitis B	6, 10, 14 weeks
Measles	Measles	9 Months

#### Kebele

Is the smallest administrative unit in Ethiopia which consists of around 2000 households. It is much similar unit to sub-district in other countries [36].

### Data quality control

Questionnaire was initially prepared in English and translated into Sidama-afoo (local language) and back to English, by language experts to ensure its consistency. Training was given for data collectors and supervisors for two days. Pre-testing was conducted on 5% of sample size in Kebeles which were not included in actual study to check consistency and any ambiguity of the questionnaire a week back of actual data collection. Then, any deviation identified during pretest was checked again and rephrased or re-adjusted accordingly. Data completeness was checked by the supervisor daily and weekly by the principal investigator for completeness and missing data.

### Data analysis

Data were entered into Epi-data version 4.4, exported and analyzed using SPSS version 22. Data coding and cleaning was conducted before formal analysis. Descriptive analysis was carried out by frequency tables, graphs and charts. Chi-square test was performed to identify association between the outcome and independent variables. 12 variables were found to have association (*P*-value < 0.05). After necessary logistic regression assumption was checked, binary logistic regression was fitted and 10 variables were found to be associated with timeliness of vaccine (*p*-value < 0.25). Then, these variables were further fitted into multivariate logistic regression analysis to control possible cofounders. Finally, only six variables were found to be associated significantly in multivariate logistic regression analysis model. Hosmer Lemeshow goodness of fit test was used to check goodness of fit of the model, and it was good fit. Binary logistic regression was done to identify candidate variables that can be fitted into multivariable logistic regression analysis model. Statistical significance of variables at final model was declared at *p*-value less than 0.05 and 95% CI of Adjusted Odds Ratio (AOR). Finally, Hosmer–Lemeshow goodness of fit test was used to estimate the goodness of fit of the adjusted final model [37]. All cases were analyzed except the non-respondent in this study.

### Multicollinearity and interaction effect

The presence of multicollinearity was checked among independent variables using Variance Inflation Factor (VIF) at cut off point of 10 [38]. Accordingly, multicollinearity between variables was found to be VIFs < 2. Moreover, the tolerance of all variables was greater than 0.1 which indicates no meaningful multicollinearity in this study.

## Results

### Socio-demographic characteristics

Table 2 presents the socio-demographic characteristics of study participants of this study. From the total of 614 study subjects, six hundred nine (609) study participants have responded questions completely making a response rate of 99.2%. Non-respondents were examined for their main reason for loss of on time vaccination and found not related with timeliness of vaccine. The mean age of study subjects was 28.2 ± 5.1 years. Furthermore, the mean age of children was 17.2 ± 3.3 months. More than half (57.0%) of children were females. From the total study participants, 242(39.7%) study participants were illiterate. Most (94.5%) of the study participants resided in rural area. Nearly all (98.4%) study participants were married. More than half (53.2%) of the study participants earn less than 9.2 USD per month. Most of (95.4%) the study participants were Sidama ethnic group. Majorities (89.5%) of the study participants were Protestants. Five-hundred (82.3%) of the study participant were housewives in this study (see Table 2).

**Table 2:** Socio demographic characteristics of the study participants (*n* = 609)

Variables	Frequency	Percentage
Age in years		
Less than 20	11	1.8
20–24	136	22.3
25–29	241	39.6
30–34	118	19.4
35–39	86	14.1
40 and more	17	2.8
Educational status		
No education	242	39.7
Primary school	222	36.5
Secondary and above	145	23.8
Area of residence		
Rural	576	94.5
Urban	33	5.5
Occupation		
Housewife	500	82.1
Farmer	13	2.2
Government employee	18	3.0
Merchant	62	10.1
Others	16	2.6
Marital status		
Married	599	98.4
Separated/Divorced	7	1.1
Widowed	3	0.5
Number of children		
Less than 5	542	89.0
5 and more	67	11.0
Family size		
Less than 5	472	77.5
5 and more	137	22.5
Average monthly income		
Less than 9.3 USD	324	53.2
9.3–18.6 USD	221	36.3
More than 18.6 USD	64	10.5

### Information about vaccination

Based on the report of Table 3, nearly all (99%) care givers had heard about vaccination and vaccine preventable diseases. Nearly all participants (91.3%) had awareness about vaccine can protect disease. In addition, most (93.6%) of the participants knew the benefit of vaccine for healthy child. Only 35(5.8%) of the participants mentioned more than five vaccine preventable diseases. On the contrary, half (50.4%) of the participants knew that the age of children to begin vaccination is at 45th day of birth. Besides, majorities nearly four out of five (78.3%) of the study participants identified the end date of child vaccination (see Table 3).

**Table 3:** Information vaccine schedule among study participants in the study setting (*n* = 609)

Variables	Frequency	Percentage
Information about vaccine/vaccine preventable diseases		
Yes	603	99.0
No	6	1.0
Vaccine protects child from illness		
Yes	556	91.3
No	53	8.7
Important for child health		
Yes	570	93.6
No	39	6.4
Responses for vaccine preventable diseases that caregivers know		
Nothing at all	14	2.3
1–2 diseases	295	48.4
3–4 diseases	265	43.5
≥ 5 diseases	35	5.8
Sessions needed for full vaccination		
One to two	8	1.3
Three	168	27.6
Four	433	71.1
Age to begin vaccination		
Just after birth	147	24.1
At one month	140	23.0
At 45 days of birth	307	50.4
At any time	15	2.5
Age to complete vaccination		
At 9 months	477	78.3
At 6 months	51	8.4
At 12 months	79	13.0
Any time in < 5 years	2	0.3
Vaccination may cause health problem		
Yes	11	1.8
No	598	98.2

### Maternal health service utilization

Table 4 presented the maternal health service utilization, two-hundred-eighty (46%) did not have history of antenatal care visit. About 22.1% of them had at least three visits during their pregnancy of the child selected for this study. More than half (59.9%) of the primary caretakers did not get postnatal care service for the current child. Among children ever born by the mothers, majorities (60.8%) of children were from 3–4 birth order. Two-third (57%) of children was born at health institutions (see Table 4).

**Table 4:** Maternal health service of women and their children in the study setting (*n* = 609)

Variables	Frequency	Percentage
Antenatal care visits		
No visit	280	46.0
≤ two visits	194	31.9
≥ three visits	135	22.1
Postnatal care obtained		
No care taken	365	59.9
≤ two times	177	29.1
Three and above	67	11.0
Sex of the child		
Male	262	43.0
Female	347	57.0
Birth order of child		
First to third	370	60.8
Fourth and above	239	39.2
Place of delivery		
Home delivery	263	43.2
Institutional delivery	346	56.8

### Proportion of timely vaccination

**Fig. 1:**
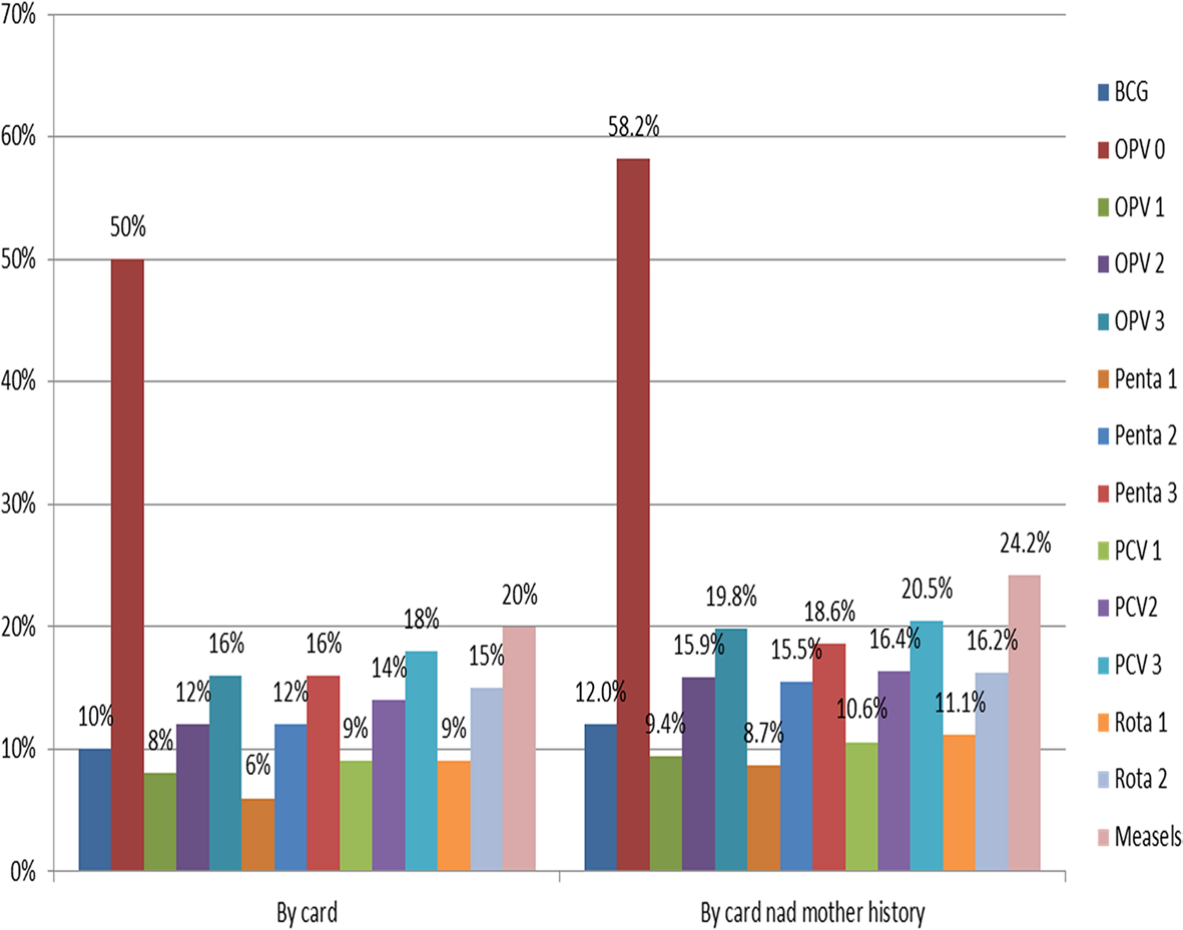
Deviation from national vaccine schedule among children of age 12–23 months, Boricha district, South Ethiopia, in 2022

Prevalence of timely vaccination was 26.8% (95%CI: 25%, 29%) in the study area. From a total of 614 study participants, 609 children aged 12–23 months gave full response making a response rate of 99.2%. Majorities 494(81.1%) of the study participants had vaccination card which indicated complete vaccination. Figure 1 highlights proportion of delayed vaccine time in this study. The most defaulted vaccine was measles (21.7%) followed by PCV_3_ (17.9%). A total of 10.8% of the children did not take BCG vaccine. Besides, OPV_1_ was the least defaulted vaccine (7.6%). Besides, measles was the most defaulted vaccine (24.2%) followed by PCV3 vaccines (20.5%). Nearly (26.8%) of the children aged 12–23 months were incompletely vaccinated by card and history from mothers of children (see Fig. 1).

### Reasons for timely vaccination

According to Table 5 description of reasons for delayed vaccination, most (45.4%) of the study participants have explained that lack of information for next dose was their main reason. Inconvenient vaccination time (39.3%) and shortage of vaccines in health institutions (32.5%) are the remain major significant problems (see Table 5).

**Table 5:** Reason of mothers for timeliness of the vaccination of their children in Boricha district, South Ethiopia, in 2022 (*n* = 382)

Reasons for out of schedule vaccination	Frequency	Percentage
Not knowing whether to come back for next dose	74	45.4
Vaccination time is inconvenient	64	39.3
Absenteeism of vaccinators	60	36.8
Due to shortage of vaccines in facility	53	32.5
Vaccination site is far-away	61	37.4
Lack of awareness on importance of vaccination	27	16.6
Fear of vaccination side-effect	19	11.6
mother sickness and being busy	24	14.7

### Factors associated with timely vaccination

Of the 12 variables that were statistically associated with timely vaccination in unadjusted analyses, six remained significant in multivariable analyses. Specifically, compared with women without formal education, women with formal education had 5.3 times more chance of having timely vaccination (AOR = 5.3, 95%CI, 2.7, 10.4). Furthermore, children whose mother without previous antenatal care had 4.3 fold higher odds of untimely vaccination than their counterparts (AOR = 4.2, 95%CI, 1.9, 9.8). The chance of late /early/ vaccination among children of age 12–23 months who was born at home was higher than their counterparts (AOR = 6.2, 95%CI, 4.0, 9.3). The likelihood of dropouts from vaccine schedule among children whose mother (caregiver) who had no postnatal care visit was 3.7 times more than their counterparts (AOR = 3.7, 95%CI, 1.2, 13.3). The odd of timeliness of vaccination among children whose mothers can not identified the time of completion of vaccine was twofold higher than their counterparts (AOR = 2.0, 95% CI, 1.2, 3.8). The chance of timely vaccination among children whose mother had three sessions needed for complete vaccination was 2.1 times higher than those of women with four sessions (AOR = 2.1, 95% CI, 1.2, 3.3) (see Table 6).

**Table 6:** Predictors of timely vaccination from logistic regression analysis data (*n* = 609)

**Study variables**	**Delay of timely vaccination**		COR (95% CI)	AOR (95% CI)
	Yes	No		
**Educational status**				
No education	98	144	6.9 (3.7,12.9)*	5.3 (2.7,10.5)**
Primary Education	52	170	3.1 (1.6, 5.9)*	3.1 (1.5,6.2)**
Secondary and above	13	132	1	1
**Previous antenatal care visits**				
Had no visit	125	155	10.1 (5.07, 20.1)*	4.3 (1.9, 9.8)**
One to two visit	28	166	2.1 (0.99, 4.5)	1.6 (0.7, 3.6)
Three or more	10	125	1	1
**Postnatal care received**				
No postnatal visit	140	225	13.3 (4.09, 43.1)*	3.7 (1.1, 13.4)**
One to two visits	20	157	2.7 (0.78, 9.5)	1.9 (0.6, 7.2)
Three or more visits	7	60	1	1
**Vaccine protects child from illness**				
Yes	128	428	1	1
No	35	18	6.5 (3.6, 11.9)*	3.5 (1.7, 7.2)**
**Important for child health**				
Yes	138	432	1	1
No	25	14	5.6 (2.8, 11.05)*	4.22 (1.9, 9.3)**
**Sessions needed for full vaccination**				
One to two	5	5	7.2 (1.7,3.9)	1.8 (0.29, 11.67)
Three	77	89	3.7 (2.5, 5.4)*	2.0 (1.23, 3.32)*
Four	81	352	1	1
**Place of delivery**				
Home delivery	123	140	6.7 (4.5, 10.1) *	6.2 (4.1, 9.4)**
Institutional delivery	40	306	1	1

Key: *COR* Crude Odds Ratio, *AOR* Adjusted Odds Ratio

^*^- *p*-value < 0.25

^**^-*p*-value < 0.05

## Discussion

Apart from measurement of total vaccine coverage, vaccine timeliness should be the key EPI indicator to measure vaccine efficacy and effectiveness. To design and implement vaccine policy and programs, contextual local studies are needed to inform the target of intervention. Hence, we have tried to highlight the prevalence and factors associated with vaccine timeliness among children of age 12–23 months in Boricha Sidama Ethiopia in 2019.

Accordingly, timeliness of vaccination among children aged 12–23 months was found to be 26.8% (95%CI: 25%, 29%) in this study. Our finding highlights that it is far behind the national target [32]. The possible explanation might be associated with the presence of vaccine misinformation, challenge in the supply chain system related to COVID-19 infection or due to fragility of the study setting because of conflict and migration. Furthermore, parents/guardians/of children might hesitate take their children for vaccination into health institutions because of fear of side effects, excessive waiting time, rumors about vaccine and fear of injection [39, 40].

When comparing our results to those of older studies, it must be pointed out that this finding is less than findings of studies done in Gondar city, Northwest Ethiopia (31.9%) [24], and Addis Ababa (55.9%), Ethiopia [29]. We have verified that differences in place of residents could result in variation of socio-demographic characteristics, access to health care and attention. In Ethiopia, urban areas historically have easier access to health services and better transportation available, whereas rural health centers may face challenges with vaccine shortages, cold chain maintenance, attrition of the health care work force, and density of clinics relative to urban areas [41]. This result goes beyond the previous report of the study North West Ethiopia (68%) [24]. The possible reason might be due to effective mentorship of vaccine program, better commitment of health extension workers and better awareness about vaccine in community.

Regarding factors associated with timely vaccination, children whose mothers were uneducated were more chance of getting timely of vaccination than their counterparts. This is consistent with findings previous studies conducted in Gondar city, Northwest, Ethiopia [42] and Tigray, North Ethiopia [43]. This might be due to low level of understanding about the benefits of vaccination, low chance of exposure and difficulty to interpret and low perceived impact of vaccine completeness. Besides, low education level can hinder the caregiver’s communication with health workers and might influence caregiver’s awareness to seek and take advantage of public health services including child vaccination. Finally, educated women may choose health care services that generate better health. This may be because education may provide greater knowledge of the health care utilization and the ability to respond to new knowledge more rapidly [44–46].

Children of women who did not attend antenatal care were more likely to default from vaccine schedule than their counterparts. This finding is similar with findings of studies conducted in Gondar city, Northwest Ethiopia [42], and Debre-Lebanon, Central Ethiopia [47]. This consistency can be explained by the fact that pregnant women who attend prenatal care can be counseled and reminded the vaccine schedule. Moreover, utilization of subsequent health service such as delivery in the health institution, postnatal care, family planning and expanded programme of immunization can be advocated at antenatal care visit.

Antenatal care visit at a health institution during pregnancy can have better chance of taking one or more tetanus toxoid vaccine which creates further opportunities to obtain adequate information to discuss vaccines and vaccine-preventable diseases and to encourage adherence to vaccine schedules [48–50]. On-time vaccination was found to be higher among children whose mothers attended postnatal care than their counterparts. This find is in line with previous study conducted in Kenya [35]. The possible explanation could be due to strong counseling about vaccine schedule and provision of vaccine card among women during postnatal visit and service integration of postnatal care with EPI. Furthermore, the fact that most caregivers are females than males indicate that males have less chance to be involved during prenatal care, child birth and postnatal care. Therefore, they might have less chance of knowledge about vaccination. Furthermore, the have more social pressure.

Mothers who gave birth at home were more likely to default from the vaccination schedule than their counterparts. A similar pattern of result was obtained the study done in the west Shewa zone [42]. The possible reason for this consistency might be due to lack of information about the benefits and time of the vaccine during childbirth and immediate postnatal care. Furthermore, women who gave birth at home might be exposed to wrong cultural perceptions and practice about disease prevention for children than vaccine. Children of women who did not know a vaccine protect children from illness were more likely to take delayed vaccine than their counterparts. This result ties well with previous study done in West Shewa, Ethiopia [42]. The possible explanation might be that, as women did not have awareness about vaccine protection against illness, they could not complete vaccines. Moreover, special preparation and planning revisit programs may be done when women are aware of complication of incomplete vaccination. A similar conclusion was reached that lack of access to information and knowledge about, by whom, where, and when children should be vaccinated were significantly associated with defaulting from completion of children vaccinated [51, 52].

Children of caregivers who did not know the importance of vaccine had more chance of taking an untimely vaccine than their counterparts. Our result was broadly in line with finding of studies done in Hawassa zuria [53], Sinana district, South East of Ethiopia [54]. The possible rationale could be due to caregivers that could not change their health-seeking behavior to vaccinate their children unless they are well informed and correct their misconception. Children whose caregivers were know that the maximum number of vaccine dose is three doses were more likely to delay from on-time vaccination than caregivers who know four doses for completing vaccine. The basic finding is consistent to the findings of previous studies [31, 55, 56]. The probable explanation could be due to the fact that lack of clear information from health providers about timing of vaccination until completion of vaccine doses.

### Limitation of the study

This study had a number of strengths. First, it addresses important public health problem through community based study yielding accurate estimate. Moreover, it filled the gap by assessing the problem in most affected rural children. An apparent limitation of the method is that it cannot prove the causal relationship between the outcome and explanatory variables. Further, this study suffers from the limitation of recalling bias.

However, we ascertained vaccination outcomes objectively from expanded programe of immunization cards and expanded programe of immunization registers of health facilities, and we restricted the analysis to children of age 12–23 months to minimize recalling bias. Another limitation of this study had could not assess distance from health facilities. Lastly, this study cannot show the separate analysis of cases who seek vaccines lately or early. Further research should be done using qualitative methods to explore more about women perceptions health provider and stakeholder opinions and contextual assessment about on-time vaccination in the study area. Besides, future studies should make disaggregate analyses for a late and early vaccine.

## Conclusion

To conclude, the proportion of timely vaccination among children was lower than the national threshold. Uneducated women, history of absence in antenatal care visit, children who gave birth at home, mothers without postnatal care visits and lack of information about the benefits of vaccination and number of sessions needed for full vaccination were statistically significant with delays in timely vaccination of children aged 12–23 months. Therefore, efforts should be strengthened to expand maternal health services. Furthermore, strong awareness creation should be intensified not only for vaccine coverage but also for on time vaccination based on national schedule.

### Public health implication

The results of this study can be a benchmark for policy makers mainly situated at regionally. They can be well informed about strengthening and integrating vaccine information into maternal health services such as antenatal, delivery and postnatal care.

### Supplementary Information

**Additional file 1.** Questionnaires.

## Data Availability

For those who are interested; the datasets of this study could be accessed from the corresponding author on reasonable request.

## Abbreviations

AOR: Adjusted Odds Ratio
CI: Confidence Interval
EDHS: Ethiopian Demographic and Health Survey
EFY: Ethiopian Fiscal Year
FMOH: Federal Ministry of Health
NGO: Nongovernmental Organization
OR: Odds Ratio
BCG: Baccilus Calmet Gullerian
