# Supplementary material for: Predictors of timeliness of vaccination among children of age 12–23 months in Boricha district, Sidama region Ethiopia, in 2019
Source: BMC Pediatr. 2023 Aug 19;23:409. doi: 10.1186/s12887-023-04234-4 (PMC10439539; doi:10.1186/s12887-023-04234-4)
Supplement: Supplementary file 1 — Additional file 1. Questionnaires. [file 12887_2023_4234_MOESM1_ESM.docx]

## Questionnaires (English version)

**Informed consent**

Dear participants! My name is-------- I am one of the data collectors for the study conducted in Boricha district about timeliness of vaccine by investigators from Hawassa University College of Medicine and Health Science. Therefore, you are chosen to participate in this study by chance.

**Objective:** The aim of the study is to determine prevalence and possible factors associated with timeliness of vaccine in Boricha district in Sidama region, Ethiopia, in 2019.

**Purpose:** The purpose of this study is to generate information about associated factors of timeliness of vaccine in the health, stakeholders and significant others to take actions based on the findings. The study will involve various factors about parent or children factors of vaccination. In order to effectively attain the objective of the research, we are requesting your help.

**Confidentiality:** Your responses will be completely confidential, any personal information given for the data collector will not be informed and transferred to other bodies, All Personal identifiers &amp; personal information will not be taken. Information will be accessed by the researcher and research assistant only. It is your full right to refuse in responding any question or all of the questions during interview. If you want to participate in any stage you can stop me. However, your honest answers to these questions will help us in better understanding of associated factors for timeliness of vaccine, so; we are requesting you to give your honest responses and Keep participation. It will take a maximum of 30 minutes to answer these questions.

**Risk and/or Discomfort:**

There is no any risk or discomfort that you will face by participating in this research except dedication of time for responding the questioner. Any personal information given for the data collector will not be informed and transferred to other bodies. Every piece of information will be kept confidentially. There is no any risk in participating in this research project.

**Benefits:**

There will be benefit for Sidama region health department in participating in this research. The findings of the study are no doubt to implement the intervention and making them aware of the problem and participant in prevention and control activities in the community through announcing the proximal and distal determinant factors for vaccine timeliness. Based on the findings of the research, generally it will help to design effective and appropriate measure for the prevention of vaccine delay and prevent vaccine preventable.

**Incentives/Payment for Participating:** There is no incentive or payment to be gained by taking part in this project.

**Contact address:** In case if you have any question you can contact investigator (Berhan Tsegaye)

Phone number ----+251967136387

Will you willing to participate please?

Yes ----Continue the interview No------Thank him/her and quit.

Date and signature------------

| **PART ONE: - SOCIO - DEMOGRAPHIC ASSESSMENT** | | | | | | | | | | | | | | |
| --- | --- | --- | --- | --- | --- | --- | --- | --- | --- | --- | --- | --- | --- | --- |
| Numbers | | \| Questions \|  \|  \| \| --- \| --- \| --- \| | | | Responses | | | | Code | | | | | |
| 101 | | \|  \| Child date of birth \| \| --- \| --- \| | | | 1. ____ Day____ Month__ Year 2. Don't know [if recorded on vaccination card, replace don't know response with date indicated on the card) | | | |  | | | | | |
| 102 | | Age of child in months | | | Months | | | |  | | | | | |
| 103 | | Sex of the child | | | 1. male 2. female | | | |  | | | | | |
| 104 | | Number of children ever born by the mother | | | 1. male 2. female | | | |  | | | | | |
| 105 | | Number of children alive | | | ___________ | | | |  | | | | | |
| 106 | | Number of total Family size in household | | | ____________ | | | |  | | | | | |
| 107 | | Birth order of this child | | | 1. 1st 2. 2nd 3. 3rd 4. 4th 5. 5th 6. 6th 7. 7th & above | | | |  | | | | | |
| 108 | | Mother's marital status | | | 1. single 2. married 3. separated 4. divorced 5. widowed | | | |  | | | | | |
| 109 | | Who is the primary caretaker of the child? | | | 1. Mother 2. Father 3. Other(specify) _____ | | | |  | | | | | |
| 110 | | What is the age of primary care taker? | | | 1. -------years  99. No response  88. I don't know | | | |  | | | | | |
| 111 | | What is educational status of caretaker? | | | 1. Illiterate 2. Read and write 3. Grade1-8 4. Grade 9-12 5. College/university | | | |  | | | | | |
| 112 | | What is the occupation of the primary caretaker? | | | 1. Housewife 2. Farmer 3. Government employee 4. merchant 5. Daily laborer 6. others, specify_______ | | | |  | | | | | |
| 113 | | What is the Ethnicity of the primary caretaker? | | | 1. Sidama 2. Wolayta 3. Oromo 4. Amhara 5. Others(specify) ----------------   99. No response  88. I don't know | | | |  | | | | | |
| 114 | | Where is her/his current area of residence? | | | 1. Rural 2. Urban   99. No response  88. I don't know | | | |  | | | | | |
| 115 | | What is the religion of the primary caretaker? | | | 1. Protestant 2. Orthodox 3. Muslim 4. Catholic 5. Other Christians 6. Non-Religious | | | |  | | | | | |
| 116 | | What is your monthly income (in birr)? | | | 1. Less than 500 ETB 2. 500-1000 ETB 3. More than 1000 birr   99. No response  88. I don't know | | | |  | | | | | |
| **PART TWO: - QUESTIONS ON VACCINATION KNOWLEDGE** | | | | | | | | | | | | | | |
| Number | | Questions | | | | Responses | | | | | | Code | | |
| 201 | | Do you heard or seen about vaccination and vaccine preventable disease? | | | | 1. Yes 2. No | | | | | |  | | |
| 202 | | If yes to above question, from where do you heard about the vaccination and vaccine preventable disease? (Multiple response possible) | | | | 1. Community members 2. Health workers at health facility 3. health extension workers 4. Radio 5. TV 6. News paper 7. Kebele administrator 8. Other government official 9. Others(specify)___________   99. No response  88. I don't know | | | | | |  | | |
| 203 | | What messages have you heard about vaccinations? (Multiple response possible) | | | | 1. about campaigns (e.g. Dates, target group) 2. Importance of routine vaccination 3. Where to get routine vaccination 4. Age to get routine vaccination 5. Return for the next doses of the routine vaccination 6. About new vaccines (pneumococcal/rotavirus vaccines) 7. Other, specify_______________ 8. No response   88. I don't know | | | | | |  | | |
| 204 | | Do you mention the benefit of vaccinating a child ? (Multiple response possible) | | | | 1. To prevent the disease 2. For specific disease 3. For child health 4. Other, specify__________   99. No response  88. I don't know | | | | | |  | | |
| 205 | | What vaccine preventable diseases do you know? | | | | 1. Measles 2. Diphtheria 3. Polio 4. Tetanus 5. Pertussis 6. Hepatitis b 7. Haemophilus influenza b 8. pneumonia 9. diarrhea 10. Tuberculosis   99=No response  88=I don't know | | | | | |  | | |
| 206 | | Do you tell me the age at which the child begins vaccination? | | | | 1. just after birth 2. four weeks after a birth 3. six weeks after a birth 4. other, specify__________   99. No response  88. I don't know | | | | | |  | | |
| 207 | | How many vaccination sessions are needed for a child to be fully protected? | | | | 1. --------------   99. No response  88. I don't know | | | | | |  | | |
| 208 | | At what age the child should complete vaccination? | | | | ____________________ | | | | | |  | | |
| 209 | | Do you think vaccination may cause health problem to the child? | | | | 1. Yes 2. No 3. don't know | | | | | |  | | |
| 210 | | Have you ever decided not to take your child to get a Vaccination? | | | | 1. Yes 2. No | | | | | |  | | |
| **PART THREE: - MATERNAL HEALTH CARE UTILIZATION** | | | | | | | | | | | | | | |
| Number | | Questions | | | | | | Responses | | | | | code | |
| 301 | | Did the mother of the baby attended antenatal care during her last pregnancy? | | | | | | 1. Yes 2. No | | | | |  | |
| 302 | | If yes, how many times did you/she attend? | | | | | | ________________ | | | | |  | |
| 303 | | Where did the mother deliver her last child? | | | | | | 1. Home  2 Relative/Neighbor's home  3 Health Post  4.HealthCenter/Hospital  5.Private or NGO Facility  6.Other specify______ | | | | |  | |
| 304 | | Did you take postnatal care for this child after delivery? | | | | | | 1. yes 2. no | | | | |  |  |
| 305 | | Where do you take the child if he/she is sick? | | | | | | 1. Health Post 2. Health center/Hospital 3. Private or NGO Facility 4. Religious or traditional   places   1. Self-treatment at home 2. pharmacy 3. Other specify______ | | | | |  | |
| 306 | | Did the mother of the child use any modern method of contraceptive ever? | | | | | | 1. yes 2. no | | | | |  | |
| PART FOUR: ACCESSEBILITY AND AVAILABILITY OF VACCINATION SERVICE | | | | | | | | | | | | | | |
| 401 | Is there any nearby health facility that provides vaccination service? | | | | | | | 1. yes 2. no | |  | | | | |
| 402 | If yes to above question which health facility is near to you? | | | | | | | 1. Health center 2. Hospital 3. Health post 4. private clinic | |  | | | | |
| 403 | What means of transportation do you usually use to come to this facility? | | | | | | | 1. Walk 2. By any transport | |  | | | | |
| 404 | if the above answer is by any transportation means does it incur you any cost | | | | | | | 1. yes 2. no | |  | | | | |
| **PART FIVE: - CHILD VACCINATION** | | | | | | | | | | | | | | |
| 501 | | | \| Does your child take any vaccination? \|  \| \| --- \| --- \| | | | | 1. Yes 2. No | | | |  | | | |
| 502 | | | \| If yes to above question, do you have a card where vaccinations are written down? \|  \| \| --- \| --- \| | | | | 1. Yes 2. No | | | |  | | | |
| 503 | | | Copy the vaccination data from the card (or from health facility register if the mother doesn't remember) | | | |  | | | |  | | | |
| Vaccine code | | | Vaccine Date Registration | | | | | | | | | | | |
|  |  |  | Vaccine type | \| DD/MM/YY \| DD/MM/YY \| DD/MM/YY \| DD/MM/YY \| DD/MM/YY \| \| --- \| --- \| --- \| --- \| --- \| \| Date of birth \| First dose \| Second dose \| Third dose \| Fourth dose \| \|  \|  \|  \|  \|  \| \|  \|  \|  \|  \|  \| \|  \|  \|  \|  \|  \| \|  \|  \|  \|  \|  \| \|  \|  \|  \|  \|  \| \|  \|  \|  \|  \|  \| \|  \|  \|  \|  \|  \| \|  \|  \|  \|  \|  \| \|  \|  \|  \|  \|  \| \|  \|  \|  \|  \|  \| \|  \|  \|  \|  \|  \| \|  \|  \|  \|  \|  \| \|  \|  \|  \|  \|  \| \|  \|  \|  \|  \|  \| | | | | | | | | | |  |
| 01 | | |  |  |  |  |  |  |  |  |  |  |  |  |
| 02 | | | BCG |  |  |  |  |  |  |  |  |  |  |  |
| 03 | | | OPV0 |  |  |  |  |  |  |  |  |  |  |  |
| 04 | | | OPV1 |  |  |  |  |  |  |  |  |  |  |  |
| 05 | | | OPV2 |  |  |  |  |  |  |  |  |  |  |  |
| 06 | | | OPV3 |  |  |  |  |  |  |  |  |  |  |  |
| 07 | | | OPV4 |  |  |  |  |  |  |  |  |  |  |  |
| 08 | | | Penta1 |  |  |  |  |  |  |  |  |  |  |  |
| 09 | | | Penta2 |  |  |  |  |  |  |  |  |  |  |  |
| 10 | | | Penta3 |  |  |  |  |  |  |  |  |  |  |  |
| 11 | | | Measles |  |  |  |  |  |  |  |  |  |  |  |
| 12 | | | PCV1 |  |  |  |  |  |  |  |  |  |  |  |
| 13 | | | PCV2 |  |  |  |  |  |  |  |  |  |  |  |
| 14 | | | PCV3 |  |  |  |  |  |  |  |  |  |  |  |
| 15 | | | Rota1 |  |  |  |  |  |  |  |  |  |  |  |
| 504 | | | Has a child had any vaccinations that are not recorded on this card? Including vaccinations given in a national vaccination day campaign? | 1. Yes 2. No   99. No response  88. I don't know | | | | | | | | | |  |
| 505 | | | If answer to above question is yes, what is the type of vaccines? | 1. vaccine given to prevent the child from measles in mass campaign 2. vaccine given to prevent the child from polio in mass campaign 3. routine vaccine 4. others, specify___________   99. No response  88. I don't know | | | | | | | | | |  |
| 506 | | | What are the reasons for defaulting from the normal schedule? If child is a defaulter) (Multiple response possible) | 1. Vaccination site is far-away 2. Vaccination time is inconvenient 3. Absenteeism of vaccinators 4. Lack of awareness on the importance of vaccination 5. Not knowing vaccination time and site 6. Not knowing whether to come back for second and third vaccination 7. fear of side effects 8. lack of transportation 9. Others | | | | | | | | | |  |
| 507 | | | Please tell me if the child had any of the following vaccinations |  | | | | | | | | | |  |
| 508 | | | A. BCG vaccination against tuberculosis that is an injection in the arm or shoulder that usually causes a scar | 1. Yes____  2. No______  99. No response  88. I don't know | | | | | | | | | |  |
| 509 | | | \| B. Polio vaccine that is drops in the mouth? \|  \| \| --- \| --- \| | 1. Yes 2. No   99. No response  88. I don't know | | | | | | | | | |  |
| 511 | | | Was the first polio vaccine given in the first two weeks after birth or later? | 1. Yes 2. No   99. No response  88. I don't know | | | | | | | | | |  |
| 512 | | | How many times was the polio vaccine given | ______________________  99. No response  88. I don't know | | | | | | | | | |  |
| 513 | | | Is pentavalent vaccination that is given by injection in the right thigh? | 1. Yes 2. No   99. No response  88. I don't know | | | | | | | | | |  |
| 514 | | | How many times Pentavalent vaccination is given? | ______________  99. No response  88. I don't know | | | | | | | | | |  |
| 515 | | | D. a PCV vaccination, that is, an injection given in the left thigh ? | 1.Yes  2.No  99. No response  88. I don't know | | | | | | | | | |  |
| 516 | | | How many times PCV vaccination is given? | _______  99. No response  88. I don't know | | | | | | | | | |  |
| 517 | | | E. Was the Rota vaccine given as a drop the same day of the second round polio drop is given? | 1. Yes  2. No  99. No response  88. I don't know | | | | | | | | | |  |
| 518 | | | How many times was the Rota vaccine given | ______________  99. No response  88. I don't know | | | | | | | | | |  |
| 519 | | | F. a measles injection that is, a shot in the arm at the age of 9 months or older – to prevent him/her from getting measles? | 1. Yes__________  2. No__________  99. No response  88. I don't know | | | | | | | | | |  |

**Thank you for your obedience and participation!!**
